# Immersion Shadowing: Nursing Student Reflections of Post-Acute and Long-Term Care (PALTC)

**DOI:** 10.1093/geroni/igaf122.1375

**Published:** 2025-12-31

**Authors:** Jinmyoung Cho, Susan Elliott, Marla Berg-Wedger

**Affiliations:** Saint Louis University, Saint Louis University, Missouri, United States; Saint Louis University School of Medicine, St. Louis, Missouri, United States; Saint Louis University, Saint louis, Missouri, United States

## Abstract

This presentation focuses on the assessment of short-term student learning outcomes, using a reflective tool as a means of both facilitating student learning as well as assessing their learning in a geriatric nursing program. Great geriatric care requires a deep understanding of the complex physical, cognitive, and psychosocial needs of older adults. As the demand for well-trained professionals in post-acute and long-term care (PALTC) increases, effective educational strategies are essential. This presentation examines an experiential learning case where the use of reflective assessment was used to evaluate short-term student learning outcomes in geriatric nursing education. As part of an experiential learning program, 163 upper-level undergraduate nursing students shadowed staff in PALTC settings to gain first hand exposure to geriatric and dementia care. The program integrated two key learning components: (1) a structured session on Age-Friendly Health System (AFHS) concepts and the 4Ms framework, and (2) direct observation and clinical experience in a PALTC facility. To assess learning, students completed pre and post knowledge assessments and engaged in structured reflection to explore their evolving perceptions of geriatric care, interprofessional collaboration, and the realities of working in PALTC settings. This session will highlight how reflective tools can both facilitate student learning and serve as a critical assessment strategy within geriatric education. Attendees will gain practical insights into designing reflective assessments that capture meaningful learning outcomes and inform program development to better prepare future healthcare professionals for geriatric practice.

